# Tendon Interposition and Ligament Reconstruction with ECRL Tendon in the Late Stages of Kienböck's Disease: A Cadaver Study

**DOI:** 10.1155/2013/416246

**Published:** 2013-03-31

**Authors:** Nazım Karalezli, Aysun Uz, Ali Fırat Esmer, Mehmet Demirtaş, Arzu Gül Taşcı, Harun Kütahya, Gürhan Ulusoy

**Affiliations:** ^1^Department of Orthopaedics and Traumatology, Meram School of Medicine, Necmettin Erbakan University, Konya, Turkey; ^2^Department of Anatomy, School of Medicine, Ankara University, Ankara, Turkey; ^3^Department of Orthopaedics and Traumatology, School of Medicine, Ankara University, Ankara, Turkey; ^4^Department of Engineering Sciences, Experimental Mechanics and Biomechanics Laboratory, Middle East Technical University, Ankara, Turkey; ^5^Department of Plastic and Reconstructive Surgery, Ankara Training and Research Hospital, Ankara, Turkey

## Abstract

*Background*. The optimal surgical treatment for Kienböck's disease with stages IIIB and IV remains controversial. A cadaver study was carried out to evaluate the use of coiled extensor carpi radialis longus tendon for tendon interposition and a strip obtained from the same tendon for ligament reconstruction in the late stages of Kienböck's disease. *Methods.* Coiled extensor carpi radialis longus tendon was used to fill the cavity of the excised lunate, and a strip obtained from this tendon was sutured onto itself after passing through the scaphoid and the triquetrum acting as a ligament to preserve proximal row integrity. Biomechanical tests were carried out in order to evaluate this new ligamentous reconstruction. *Results*. It was biomechanically confirmed that the procedure was effective against axial compression and distributed the upcoming mechanical stress to the distal row. *Conclusion*. Extensor carpi radialis longus tendon has not been used for tendon interposition and ligament reconstruction in the treatment of this disease before. In view of the biomechanical data, the procedure seems to be effective for the stabilization of scaphoid and carpal bones.

## 1. Introduction

Kienböck's disease is a painful idiopathic disorder of the wrist in which roentgenograms show avascular necrosis of the carpal lunate. If remains untreated, the disease usually results in fragmentation of the lunate, collapse with shortening of the carpus and arthritis throughout the proximal carpal area [1]. 

Numerous conservative and operative treatment modalities have been advocated for Kienböck's disease ranging from immobilization to wrist arthrodesis. Some of the more common operations are limited carpal fusions [2–4] resection arthroplasty and prosthetic replacement with various materials like tendon, fascia, silicone, pyrocarbon prosthesis, and so forth [5–9], joint levelling procedures [10, 11], lunate revascularization [12–14], and proximal row carpectomy (PRC) [15, 16]. 

In the late stages of the disease when the lunate is unsalvageable and needs to be excised, resection arthroplasty, PRC, limited carpal fusions, and wrist arthrodesis are the treatment options. PRC and limited carpal fusions require preservation of hyaline cartilage in the lunate and scaphoid fossae of the radius, respectively, and otherwise total wrist fusion may be required [17]. 

Fragmented lunate excision and prosthetic replacement is an alternative technique in the late stages. However, excision of the lunate causes complete loss of scapholunate and lunotriquetral interosseous ligament integrity which should be reconstructed at the time of lunate replacement in order to preserve proximal row integrity and carpal height and to avoid scaphoid flexion [18]. Scapholunate ligament injury and scaphoid malrotation are etiologic factors for SLAC and SNAC wrist, respectively [18].

The described surgical technique uses ECRL tendon strip for proximal row integrity and coiled ECRL tendon for anchovy in cadaver specimens. Biomechanical tests were carried out in order to evaluate this new ligamentous reconstruction.

## 2. Methods

**Figure 1:**
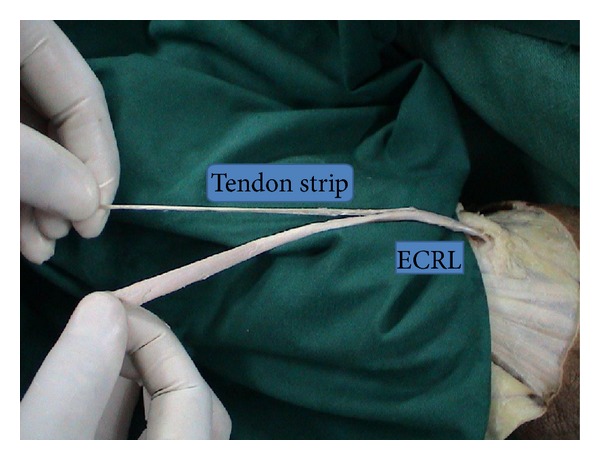
The strip obtained from ECRL and the remaining part of the tendon which will be folded into a coil.

**Figure 2:**
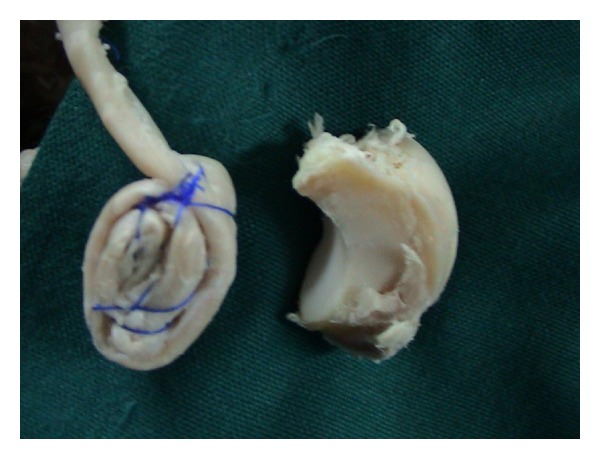
The coiled ECRL tendon and an excised lunatum.

**Figure 3:**
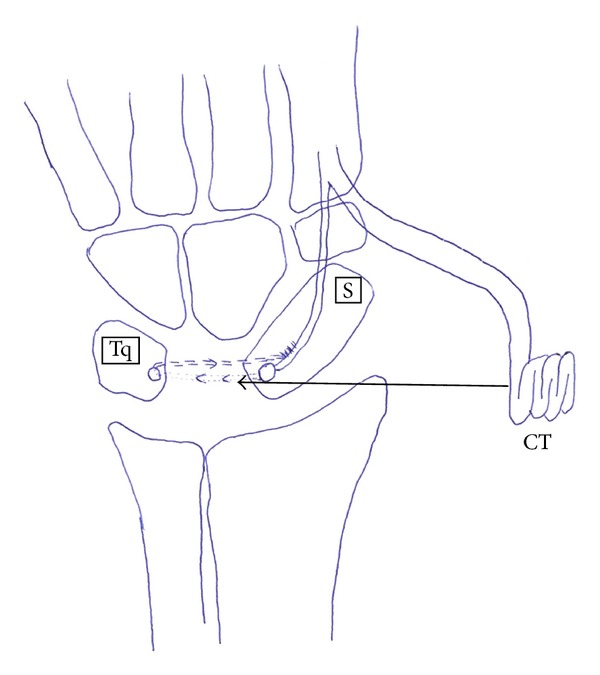
Schematic presentation of ligament reconstruction with tendon interposition arthroplasty (CT: coiled tendon; S: scaphoid; Tq: triquetrum; Ts: tendon strip for ligament reconstruction).

Six cadaver limbs which were cut 10 cm distal to the elbow were dissected. First, a transverse incision was made in the dorsal aspect of the wrists. Extensor pollicis longus (EPL) tendon, wrist, and finger extensors were dissected out and protected. Wrist joint capsule was opened in U shape, and the lunatum was identified and excised. Brachioradialis, extensor carpi radialis longus (ECRL), and extensor carpi radialis brevis (ECRB) tendons were found in the middle third of forearm. ECRL was cut at this level from the musculotendinous junction. It was retrieved distally with its distal insertion to the second metacarpal left intact. A tendon strip was obtained from the tendon (Figure 1). The remaining part of the tendon was folded into a coil and held firmly by transfixing sutures of prolene (Figure 2). Then, two holes of 2.7 mm were drilled to the triquetrum and to the proximal scaphoid, and the tendon strip obtained from ECRL tendon was passed through these holes. The coiled tendon was inserted into the cavity of excised lunate, and the tendon strip was sutured tightly onto itself over the coiled tendon (Figure 3). Then, the capsule was closed end to end with prolene. 

**Figure 4:**
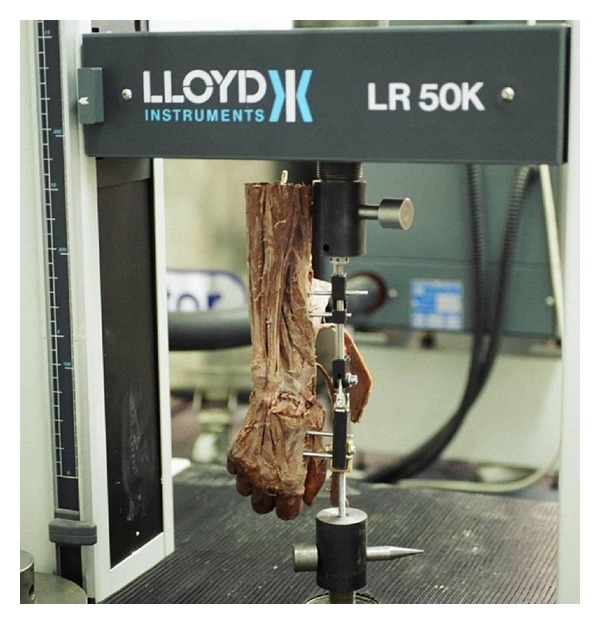
Loading machine and external fixator with a cadaver extremity positioned and ready for testing.

Then, vertical compression tests were performed through custom made external fixator associated with a Lloyd LR 50 K Standard Testing Machine (Southampton, UK). 1000 N load cell was inserted into the testing machine. Loading rate was decided as 1 mm/min. The cadaver wrists were loaded upto 500 N (=50 kg) (Figure 4).

Pressure sensitive films (Fuji Prescale Film, USA) were used during tests. These films contain pressure sensitive balloons. The amount of exploded balloon number is directly proportional with the load applied to the film.

Sensitive films were cut to match the joint surface area. Pressure sensitive films were covered with gelatine to maintain watertightness. Joint surfaces where these sensitive films had been placed were also dried.

**Figure 5:**
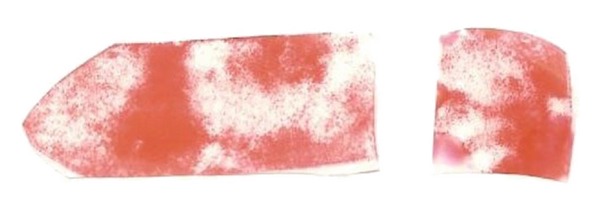
Pressure sensitive films. Bigger one is from radioscaphoid and the smaller one is from scaphocapitate joint.

Larger films were located between scaphoid and radius, and smaller ones were positioned between scaphoid and capitatum (Figure 5). The rough textures of sensitive films were set to face each other. During preparation and locating processes, special care was taken in order not to apply finger pressure on films. After the test, films were carefully pulled out and put in envelopes avoiding excess light exposure. 

**Figure 6:**
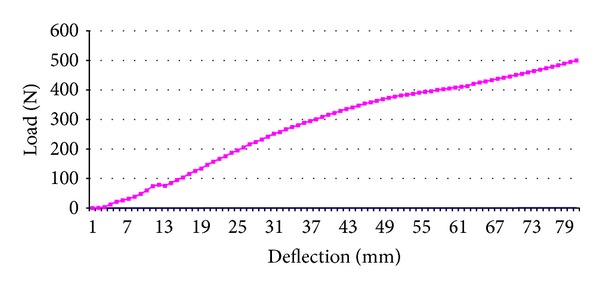
Graph of load deflection under compression.

All sensitive films were scanned, digitized, and transferred to computer with their calibration scale. Pressure distributions were determined with Lucia v. 4.21 Software (Nikon Co., Japan). In order to calculate the modulus of elasticity, the load deflection curves were obtained (Figure 6). Lastly, the load magnitudes and percentages of pressure distribution were calculated.

## 3. Results

The coiled ECRL tendon was bulky enough to fill the cavity formally occupied by the lunatum (Figure 2). As well as that, the strip obtained from the ECRL tendon had enough length to pass through the scaphoid and the triquetrum and to be sutured onto itself (Figure 1). 

### 3.1. Biomechanical Test Results

The distribution of exploded balloon among small and large pressure sensitive films was calculated (Table 1). Huge percent of the applied load was detected by large sensitive films which were located between scaphoid and radius. Load magnitudes and percentages of load transmission were also calculated (Table 2). There was an approximately 27% to 73% load distribution among scaphocapitate and radioscaphoid joint surfaces, respectively. 

Load deflection curve confirmed that the coiled tendon and the tendon strip between scaphoid and triquetrum resisted the compression force and was effective in carrying and distributing the upcoming loads to the distal row (Figure 6).

The statistical evaluation was done with Graphpad Prism Software v. 4.0 (San Diego, CA, USA). 

## 4. Discussion

Definite treatment of Kienböck's disease with stages IIIB and IV characterized by progressive carpal collapse, scaphoid malrotation, and osteoarthritic changes remains controversial. The ideal procedure should relieve the pain and maintain normal wrist function as much as possible.

Excision of the lunate bone in the late stages of Kienböck's disease relieves the pain. To maintain grip strength, increase range of motion, and prevent carpal collapse, this space has been filled with tendon, fascia, and various materials such as steel, ivory, silicone, and pyrocarbon [5–9].

Localized foreign body synovitis or wear particle synovitis in response to silicone prostheses has been reported. On the other hand, the silicone prosthesis collapse rates up to 22% and scaphoid rotation have been reported. These abnormal biomechanics over a prolonged period produce a chronic irritation at the wrist [5, 7, 8]. According to Alexander et al., the results of silicone replacement arthroplasty (SRA) in the treatment of stage 3 Kienböck's disease are unsatisfactory in early postoperative period which also deteriorate with time [5]. Kato et al. compared silicone implant with coiled palmaris longus tendon in 32 cases treated by excisional arthroplasty. Silicone implant was reported to be more effective than palmaris longus tendon replacement in terms of further carpal collapse. However, postoperative progression of osteoarthritic changes or subluxation of prosthesis in the advanced stages of carpal collapse was also stressed [7]. It is also clear that neither silicone nor a palmaris tendon ball can maintain proximal row integrity. 

Limited arthrodesis is another treatment option in the late stages. As symptomatology at stage 3 was linked mainly to rotatory subluxation of the scaphoid, Watson et al. proposed a limited wrist arthrodesis between scaphoid, trapezium, and trapezoid (STT). They concluded that STT arthrodesis alone might suffice to bear the wrist load and reported relief of pain in 16 cases treated by STT artrodesis with or without silicone lunate arthroplasty [1]. In a more recent study, Rogers and Watson documented painful radial styloid impingement after a successful STT artrodesis and recommended a partial radial styloidectomy as a routine procedure during STT artrodesis [19]. On the other hand, Minami suggested that radial styloidectomy would not be effective in preventing osteoarthritis after STT artrodesis [2]. Moreover, the STT arthrodesis was stressed to deteriorate wrist motion significantly [1, 2, 4].

PRC is another treatment option for unsalvageable lunate. However, PRC and limited carpal fusions require preservation of hyaline cartilage in the lunate fossa and scaphoid fossa of the radius, respectively. If collapse has persisted for too long, these procedures cannot be utilized due to the degradation of cartilage at these regions [17].

In a recent study, lunate replacement arthroplasty with pyrocarbon prosthesis and double bundle split flexor carpi radialis (FCR) tendon graft for rotational stabilization of the scaphoid was described. The most likely complication was described as fracture of the bone tunnels in either scaphoid or triquetrum because of creating two bone tunnels in a small bone. Also, drilling two tunnels can induce an iatrogenic Preiser's disease. Inappropriate size of the implant may lead to imbalanced load transfer. Oversizing would result in capitolunate and lunate fossa wear whereas an undersized implant would lead to radioscaphoid wear. Loss of graft fixation and/or complete implant extrusion are other probable complications.

**Figure 7:**
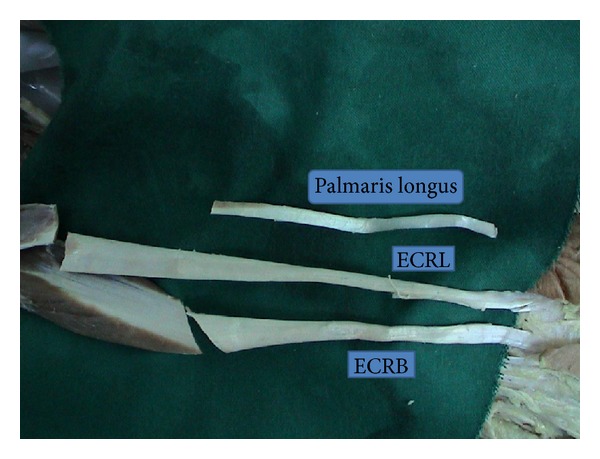
ECRL, ECRB, and palmaris longus tendons.

In this study, for an alternative treatment in the late stages of this disease, lunatum was excised to relieve pain, and an autogenous material-ECRL tendon was used to fill the space. A strip obtained from the same tendon was passed through the scaphoid and the triquetrum to act as a ligament to prevent scaphoid rotation and to preserve proximal row integrity. ECRL tendon is preferred as it is longer and thicker than palmaris longus (Figure 7), which would prevent further carpal collapse more effectively. The extensor carpi radialis brevis tendon which lies in the same anatomic region and has similar morphologic properties was not utilized, because of its centralizing effect for the wrist. 

Subluxation, oversizing, or undersizing of the graft will not be a problem in this technique. Subluxation can be avoided by suturing the graft to the volar capsule and tendon strip. An accurately sized graft may be obtained easily by reducing or including more tendon from musculotendinous intersection of ECRL. 

Sacrificing one of the wrist extensors may impair wrist function and strength. However, King et al. treated thumb carpometacarpal arthritis with a combination of trapezium excision, ligament reconstruction, and tendon interposition with ECRL tendon. The authors reported that function and strength of the thumb had improved, and wrist motion and strength was not affected after sacrificing ECRL tendon [20]. 

The ideal procedure for the treatment of Kienböck's disease should preserve biomechanics of the wrist as much as possible. In order to evaluate this, load transmission percentage at the scaphocapitate joint after the operation was measured. It was approximately 27% of the total load (Table 2) and was similar to the normal load mechanics of the scaphocapitate joint which had been reported as 28% [13]. Also, load deflection curve confirmed that coiled tendon and ligament reconstruction resisted the compression force. The procedure was effective in carrying the vertical compression loads and distributing the upcoming loads to the distal row (Figure 7). According to the test results, there was an approximately 27% to 73% load sharing among scaphocapitate and radioscaphoid joint surfaces, respectively.

In this study, use of coiled ECRL tendon and a tendon strip obtained from the tendon was proposed for the late stages of Kienböck's disease. Biomechanical data suggested that wrist stability will be achieved. The other advantages of this recommended technique may be that it can be revised by other standard operations and it is cost effective. Also, use of autogenous material prevents chronic irritation findings that may be observed in silicone implant reconstructions.

However, this is not a clinical study, and further investigations on a larger number of clinical cases are necessary to determine the efficacy of this technique. Also, we do recommend this technique only in late stages of Kienböck's disease when the lunate is evaluated unsalvageable by the surgeon.

## Figures and Tables

**Table 1:** The distribution of exploded balloon among small and large pressure sensitive films.

Specimen number	1	2	3	4	5	6
Small film	2889	1344	3374	777	4592	495
Large film	7752	4233	7619	1950	10432	1816

**Table 2:** Load absorbed by the films and load transmission percentages.

Specimen number	1	2	3	4	5	6	Average	SD	SE
Load magnitudes (Newton)									
Small film	135.7	120.5	153.5	142.5	152.8	107.1	135.3	18.46	7.5
Large film	364.3	379.5	346.5	357.5	347.2	392.9	364.7	18.46	7.5
Load transmission percentage (%)									
Small film	27	24	31	28	31	21	27	4	1,6
Large film	73	76	69	72	69	79	73	4	1,6
